# Phage–Bacteria Interactions in Potential Applications of Bacteriophage vB_EfaS-271 against *Enterococcus faecalis*

**DOI:** 10.3390/v13020318

**Published:** 2021-02-19

**Authors:** Gracja Topka-Bielecka, Bożena Nejman-Faleńczyk, Sylwia Bloch, Aleksandra Dydecka, Agnieszka Necel, Alicja Węgrzyn, Grzegorz Węgrzyn

**Affiliations:** 1 Department of Molecular Biology, University of Gdansk, Wita Stwosza 59, 80-308 Gdansk, Poland; gracja.topka@phdstud.ug.edu.pl (G.T.-B.); bozena.nejman-falenczyk@ug.edu.pl (B.N.-F.); aleksandra.dydecka@phdstud.ug.edu.pl (A.D.); agnieszka.necel@phdstud.ug.edu.pl (A.N.); 2 Laboratory of Phage Therapy, Institute of Biochemistry and Biophysics, Polish Academy of Sciences, Kładki 24, 80-822 Gdansk, Poland; sylwia.bloch@ug.edu.pl (S.B.); alicja.wegrzyn@biol.ug.edu.pl (A.W.)

**Keywords:** bacteriophage development, phage therapy, phage–bacteria interactions, bacterial biofilm, toxicity

## Abstract

Phage therapy is one of main alternative option for antibiotic treatment of bacterial infections, particularly in the era of appearance of pathogenic strains revealing resistance to most or even all known antibiotics. *Enterococcus faecalis* is one of such pathogens causing serious human infections. In the light of high level of biodiversity of bacteriophages and specificity of phages to bacterial species or even strains, development of effective phage therapy depend, between others, on identification and characterization of a large collection of these viruses, including understanding of their interactions with host bacterial cells. Recently, isolation of molecular characterization of bacteriophage vB_EfaS-271, infecting *E. faecalis* strains have been reported. In this report, phage–host interactions are reported, including ability of vB_EfaS-271 to infect bacteria forming biofilms, efficiency of eliminating bacterial cells from cultures depending on multiplicity of infection (m.o.i.), toxicity of purified phage particles to mammalian cells, and efficiency of appearance of phage-resistant bacteria. The presented results indicate that vB_EfaS-271 can significantly decrease number of viable *E. faecalis* cells in biofilms and in liquid cultures and reveals no considerable toxicity to mammalian cells. Efficiency of formation of phage-resistant bacteria was dependent on m.o.i. and was higher when the virion-cell ratio was as high as 10 than at low (between 0.01 and 0.0001) m.o.i. values. We conclude that vB_EfaS-271 may be considered as a candidate for its further use in phage therapy.

## 1. Introduction

*Enterococcus faecalis* is one of two enterococcal species (apart from *E. faecium*) which are responsible for significant medical problems due to nosocomial infections caused by them [1,2]. In fact, when considering sepsis and nosocomial infections, enterococci were reported at the third place among all bacterial species causing these diseases [2,3,4,5]. As much as 25–50% of fatal cases among patients in hospitals are related to infections by these bacteria [6,7,8,9,10,11]. *E. faecalis* has been reported to develop antibiotic resistance frequently [2,12]. Moreover, this species can form biofilms which impedes efficient treatment of infections as antibiotic penetration through biofilm structures is often severely impaired [13]. In this light, development of alternative therapies of *E. faecalis*-caused infections appears to be necessary.

Phage therapy, i.e., the use of bacteriophages to combat bacterial infections, is one of possible alternatives to antibiotic therapy [14]. However, development of this therapeutic option requires isolation and characterization of a large collection of bacteriophages [15]. Phages are usually specific for not only bacterial species, but also to specific strains. This might be considered as and advantage of phage therapy, since only pathogenic bacterial strains can be infected and killed, leaving natural microbiota unaffected. On the other hand, such specificity makes it necessary to collect various bacteriophages and include them into a library consisting of well-characterized viruses which can be employed in treatment of infected humans [14,15].

Although many bacteriophages infecting *E. faecalis* have been described (as summarized previously [16]), it is evident that extending such collections is crucial [14,15]. In fact, biodiversity of phages specific to bacteria belonging to this species is relatively high, making it possible to obtain a collection containing effective antibacterial agents to treat infections caused by different *E. faecalis* strains [17,18,19,20,21,22,23,24,25]. In our previous work, we have isolated and characterized an *E. faecalis*-specific bacteriophage vB_EfaS-271 [26]. This phage has been classified as a member of *Siphoviridae* family, having a dsDNA genome of about 40 kb. It is a virulent phage, revealing a short (8 min) latent period and average burst size of 70 phages per cell under optimal laboratory conditions (37 °C, rich bacteriological medium) [26]. In this work, we aimed to characterize vB_EfaS-271-host interactions in the light of potential application of this virus in phage therapy, and to test toxicity of vB_EfaS-271 to mammalian cells in assessment of its safety to treat patients.

## 2. Materials and Methods

### 2.1. Bacteria, Media, and Growth Conditions

The *E. faecalis* 271 strain was isolated from a urine sample of patient of Specialist Hospital of St. Wojciech in Gdansk (Poland) [27,28]. For all experiments, *E. faecalis* 271 was cultured in liquid Tryptic Soy Broth (TSB; BTL Company, Łódź, Poland) with aeration at 37 °C on a rotary shaker at the rate of 200 rpm. Tryptic Soy Agar (TSA; BTL Company, Łódź, Poland), supplemented with 1% glucose (Polish Chemical Reagents, Gliwice, Poland), was used as a solid medium. Petri dishes with *E. faecalis* 271 were incubated at 37 °C for 20 h. 

### 2.2. Propagation of Phage vB_EfaS-271

Bacteriophage vB_EfaS-271 was isolated from urban sewage (Gdansk Wastewater Treatment Plant, Poland) and deposited in the collection of phage strains of the Department of Molecular Biology of the University of Gdansk [26,28]. To obtain purified vB_EfaS-271 particles, *E. faecalis* 271 host was cultivated in the liquid TSB medium at 37 °C to an OD_600_ of 0.3. Phage stock solution was added to the bacterial culture at a multiplicity of infection (m.o.i.) of 0.1. The mixture was incubated at 37 °C for 1.5 h with shaking. After lysis of host bacteria, the obtained vB_EfaS-271 particles were treated with 4% chloroform (Chempur, Piekary Śląskie, Poland), and the debris of *E. faecalis* 271 cells were removed by centrifugation (4000× *g*, 10 min, 4 °C). The supernatant containing virions of vB_EfaS-271 was collected and concentrated in the presence of 10% polyethylene glycol 8000 (PEG8000; EPRO, Władysławowo, Poland) for 20 h at 4 °C. To recover virions, the overnight mixture was centrifuged (8000× *g*, 20 min, 4 °C), the supernatant was discarded, and the phage pellet was gently suspended in TM buffer (10 mM Tris-HCl, 10 mM MgSO_4_; pH 7.2). After chloroform extraction of the suspension, the number of plaque-forming units per mL (PFU/mL) was determined as described in Section 2.3.

### 2.3. Phage Titration

To determine phage titer of a suspension, the double overlay plaque assay was used. For base plate preparation, standard Petri dishes (Alchem, Torun, Poland) were filled with 25 mL of the TSA broth. The top layer was prepared by mixing of 2 mL of the TSB medium, supplemented with 0.4% agarose (Hispanagar, Burgos, Spain), with 1 mL of an overnight *E. faecalis* 271 culture. Then, the mixture was poured onto solid TSA medium to make double-layer plates. Serial 10-fold dilutions of a vB_EfaS-271 phage suspension were prepared in TM buffer (10 mM Tris-HCl, 10 mM MgSO4; pH 7.2), and 5 μL of each dilution was spotted onto the surface of the top agar. Fallowing overnight incubation of plates at 37 °C, number of plaque forming units (PFU) were counted, and the phage titer (in PFU/mL) was calculated. 

### 2.4. Determination of Number of Colony Forming Units

To estimate a titer of bacterial culture, in colony forming units per mL (CFU/mL), 100 µL samples were collected after 0, 1, 3, 6, and 24 h of cultivation of vB_EfaS-271-infected host cells. Serial 10-fold dilutions were prepared in 0.85% sodium chloride (Chempur, Piekary Śląskie, Poland), and 40 µL of each sample were spread onto TSA plates. After overnight incubation at 37 °C, number of viable *E. faecalis* 271 cells was calculated on the basis of counted colonies. 

### 2.5. Formation and Assessment of Biofilm Formed on Catheters

Sterile silicone Foley catheters CH-18 (BARD, Covington, GA, USA) were prepared as described previously [29,30]. Briefly, catheters were fragmented into 15-mm long segments, followed by cutting in half lengthwise to expose the interior surface. Such catheter pieces were placed in sterile 24-well plates and covered with 1 mL of a fresh *E. faecalis* 271 culture in TSB (OD_600_~0.1), containing approximately 10^8^ bacterial cells. Next, plates were incubated for 24 h at 37 °C for biofilm formation. Then, the medium with planktonic bacterial cells was removed from each well, and catheter segments were transferred to new wells and washed twice with sterile 0.85% NaCl. Catheter pieces covered with bacterial biofilm were exposed to 1 mL of vB_EfaS-271 phage suspension or 1 mL of TM buffer (control) for 3, 6, or 24 h. Three different phage-bacterial cell ratios (multiplicity of infection, m.o.i.) were tested: 0.0001, 0.01, and 10. After such incubation, liquid contents of the wells were removed, and catheter segments were again washed with sterile 0.85% NaCl. Catheter segments were aseptically transferred to Eppendorf tubes containing 0.4 mL of 0.85%, vortexed for 30 s, and put into an ultrasonic bath for 3 min at room temperature and 35 kHz frequency to detach bacterial cells. To quantify colony forming units per mL (CFU/mL), serial dilutions of bacterial suspensions were prepared in sterile 0.85% NaCl and spread onto TSA medium plates accordingly to the procedure described in Section 2.4. 

### 2.6. Assessment of vB_EfaS-271 Phage Cytotoxicity

The safety and the impact of purified phage particles on viability of mouse embryonic fibroblasts (BALB/c3T3 clone 31) were measured commercially, using the Neutral Red Uptake (NRU) cytotoxicity test, by the Animal Research Facility at Medical University in Łódź (Poland). This assay provides a quantitative estimation of the number of viable cells in a culture. The NRU cytotoxicity test was performed in accordance with the standard ISO 10993-5, using the cell line BALB/c3T3 clone 31, obtained from ATCC (Manassas, VA, USA) collection cat. No 62485414. Briefly, cells were grown in Dulbecco’s Modified Eagle’s Medium (Biowest, Riverside, MO, USA) containing 20% newborn calf serum (PAN-Biotech, Aidenbach, Germany), 2 mM glutamine (Biological Industries, Kibbutz Beit-Haemek, Israel), 1% sodium pyruvate (Biological Industries, Kibbutz Beit-Haemek, Israel), 100 units/mL of penicillin, and 100 mg/mL of streptomycin (Biowest, Riverside, MO, USA). For the assay, mammalian cells were seeded into 96-well plates at density of 10^4^ cells per well and cultured for 24 h at 37 °C and 5% CO_2_. After 24 h incubation, medium was removed from all wells, and cells were exposed to 200 μL of the mix solution containing culture medium and either vB_EfaS-271 phage particles (in two different concentrations, 10^4^ or 10^9^ PFU/mL) or 1 × 10^8^ *E. faecalis* 271 cells or both (m.o.i. 0.0001 or 10). In addition, 0.01% Triton X-100 or culture medium were used as positive and negative control, respectively. Plates were kept overnight in an incubator (37 °C, 5% CO_2_). Following 24 h incubation, the mix solution was removed and BALB/c3T3 cells were washed with 150 µL of PBS and treated with neutral red dye solution (0.005%) which efficiently penetrates membranes of viable cells. After 3 h incubation in the dark at 37 °C, the solution was removed, and cells were washed with 150 µL of PBS. Then, 100 µL solution containing 50% ethanol and 1% glacial acetic acid in water was added to each well to liberate the dye. Amount of released neutral red (proportional to the amount of viable cells) was measured as A_540_ in a microplate reader. The cell viability was expressed as a percentage of the control values (cells treated solely with culture medium). The experiment was done in triplicate. According to the PN-EN ISO 10993-5:2009 standard, the tested solution is considered cytotoxic if the cell viability is below 70%. Numbers of phage particles per mL (PFU/mL) and viable *E. faecalis* 271 cells per mL (CFU/mL) were quantified during this assay accordingly to the procedures described in Section 2.3 and Section 2.4, respectively. 

### 2.7. Microscopic Analyses

Control and phage- and/or bacteria-treated BALB/c3T3 cells were examined for morphological changes and cell growth inhibition under light microscope with phase-contrast (OptaView 7 software, Opta-Tech, Warsaw, Poland). Changes in cell morphology upon treatment with tested compounds were photographed after 24 h.

### 2.8. Assessment of Appearance of Phage-Resistant Bacteria

To estimate the percent of *E. faecalis* 271 colonies resistant to phage vB_EfaS-271, number of bacterial survivors in 24-h samples of phage-infected bacterial cultures (at different m.o.i.), were tested. In the first step, all isolated colonies were cultured in liquid TSB medium (BTL Company, Łódź, Poland) to OD_600_ of 0.1 at 37 °C with shaking. Then susceptibility of each isolate to vB_EfaS-271 infection was determined by the spot test. The presence of bacterial colonies after treatment with phage vB_EfaS-271 indicated an emergence of host resistance to the phage.

### 2.9. Statistical Analysis

Comparisons of two average values were performed by using Student’s *t*-test. Significant differences were marked by asterisks when *p* < 0.05 (∗), *p* < 0.01 (∗∗), or *p* < 0.001 (∗∗∗).

## 3. Results

### 3.1. Efficiency of Phage Treatment of Biofilms Formed on Catheters

**Figure 1:**
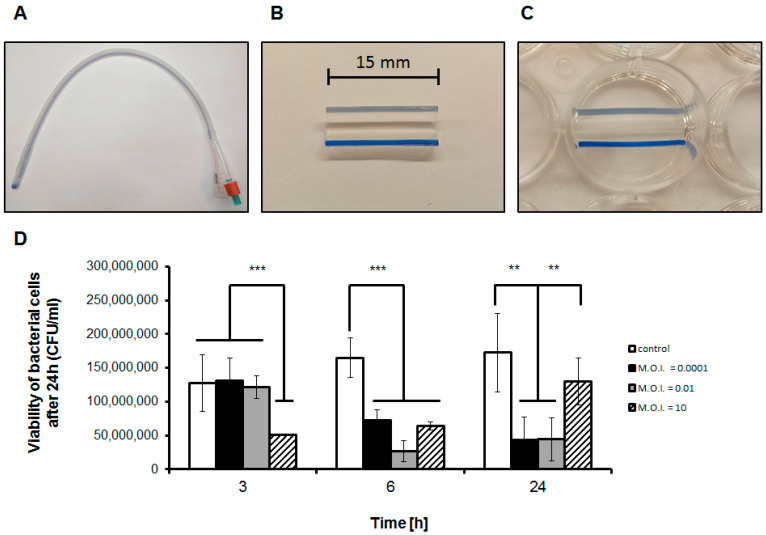
Use of vB_EfaS-271 phage against *E. faecalis* 271 biofilm formed on the catheter. (**A**) Foley silicone catheter. (**B**) A 15-mm long catheter tubing cut in half. (**C**) The catheter piece placed in the sterile 24-well plate. (**D**) Number of viable *E. faecalis* 271 cells per 1 mL (CFU/mL), quantified after 3, 6, and 24-h long exposure to vB_EfaS-271 phage added to m.o.i. of 0.0001 (closed columns), 0.01 (gray columns), or 10 (hatched columns), in relation to phage-untreated cells (open columns). Mean values from three indepenent experiments are shown with error bars indicating SD. Statistically significant differences are marked by asterisks (where ** indicates *p* < 0.01, and *** indicates *p* < 0.001).

Formation of biofilms on catheters by *E. faecalis* is considered as a serious medical problem, particularly due to enhanced resistance of bacteria included in these structures to antibiotics [13]. Therefore, we have tested efficiency of treatment of *E. faecalis*-formed biofilms by phage vB_EfaS-271. Foley catheters were cut and prepared as described in Section 2.5 and visualized in Figure 1A–C. Formation of *E. faecalis* biofilm was followed by infection with vB_EfaS-271 at different m.o.i. (0.0001, 0.01, or 10). Following 3, 6, and 24 h incubation at 37 °C, numbers of viable bacterial cells were estimated. 

As depicted in Figure 1D, infection with vB_EfaS-271 at m.o.i. 10 caused rapid (after 3 h) significant decrease in the number of viable *E. faecalis* cells. When lower m.o.i. ratios (0.0001 or 0.01) were applied, longer time (6 h) was required to observe considerable effects of the bacteriophage on viability of host bacteria. After 24 h treatment, significant reduction in the number of viable bacteria relative to control experiment (phage-untreated cells) was still observed, however, survivability of *E. faecalis* under conditions of m.o.i. 10 was considerably higher than that detected in samples where m.o.i. 0.0001 or 0.01 was employed. This unexpected result might be explained by more rapid selection of *E. faecalis* phage-resistant mutants at higher m.o.i. (see Discussion for details). Nevertheless, results presented in Figure 1 indicated that vB_EfaS-271 is efficient in decreasing the number of viable *E. faecalis* cells in biofilms formed on catheters.

### 3.2. Assessment of Toxicity of vB_EfaS-271 Phage Particles to Mammalian Cells and Effects of This Phage on E. faecalis Co-Cultured with These Cells

To test safety of phage vB_EfaS-271 to mammalian cells, we have assessed viability of mouse embryonic fibroblasts (BALB/c3T3 clone 31) after treatment with this phage. In addition, we tested effects of vB_EfaS-271 on its bacterial host co-cultured with these mammalian cells. 

**Figure 2:**
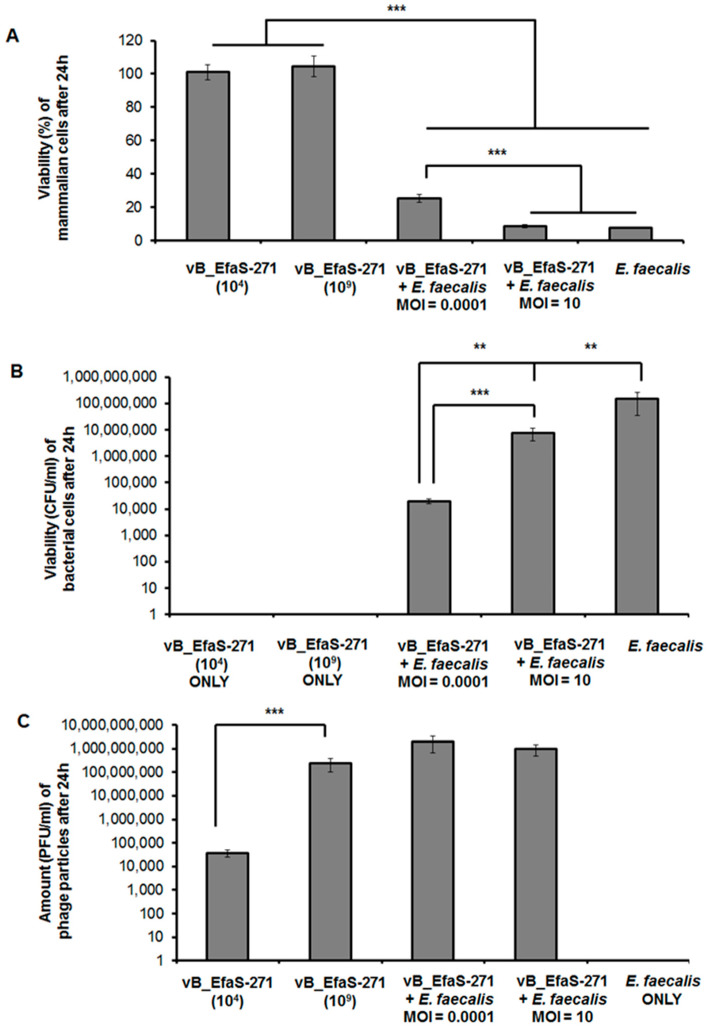
Cytotoxicity, efficacy, and stability of purified vB_EfaS-271 phage particles assessed during *E. faecalis* 271 treatment of mammalian cells. (**A**) Viability of Balb/3T3 cells exposed to different concentrations of phage vB_EfaS-271 and/or *E. faecalis* 271 for 24h. Phage suspension was tested in two different concentrations (10^4^ or 10^9^ PFU/mL) or at m.o.i. of 0.0001 or 10. (**B**) Number of viable *E. faecalis* 271 cells per 1 mL (CFU/mL) quantified after 24 h of phage treatment. (**C**) Number of phages per 1 mL (PFU/mL) assessed after 24 h after addition of phage suspension to the culture. Due to the nature of the experiments testing only bacterial cells (CFU/mL) or phage particles (PFU/mL), panels B and C lack corresponding columns (marked as “OLNY”; in these cases, the analyses were not possible). Mean values from three independent experiments are shown with error bars indicating SD. Statistically significant differences are marked by asterisks (where ** indicates *p* < 0.01, and *** indicates *p* < 0.001).

**Figure 3:**
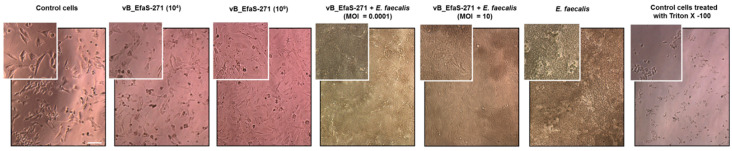
Changes in Balb/3T3 cell morphology after exposure to vB_EfaS-271 phage and/or *E. faecalis* for 24 h. Exterior panels show control cells photographed after addition of the culture medium or Triton X-100 (0.01%). Images were made using the light microscopy with phase-contrast. Bar represents 100 μm. For a better comparison, a selected part of each photo is enlarged (left top corner).

When BALB/c3T3 fibroblasts were cultured for 24 h in the presence of either 10^4^ or 10^9^ phages per mL, no significant changes in the number of viable cells, relative to non-treated cultures (viability of cells in such cultures was assumed to be 100%), were detected (Figure 2A). This result strongly suggested a lack of deleterious effects of the tested phage on mammalian cells. Such interpretation was corroborated by observations of these cells under microscope, where no morphological changes could be observed after treatment with vB_EfaS-271 in comparison to control (untreated) cells (Figure 3). 

Addition of *E. faecalis* cells (up to 10^8^ CFU/mL) to cultures of BALB/c3T3 fibroblasts resulted in a significant decreased of viability of the latter cells after 24-h incubation (Figure 2A). Simultaneous treatment with vB_EfaS-271 partially restored viability of fibroblasts when m.o.i. of 0.0001, but not m.o.i. of 10, was used (Figure 2A). These results are compatible with determination of number of *E. faecalis* cells under the same experimental conditions which was considerably higher at m.o.i. of 10 than at m.o.i. of 0.0001 (Figure 2B). On the other hand, phage titers were comparable after such 24-h incubation irrespective of the used m.o.i. (either 0.0001 or 10) at the beginning of experiment (Figure 2C). Again, effects of *E. faecalis* and mixtures of this bacterium and phage vB_EfaS-271 were confirmed in microscopic observations (Figure 3).

These results indicate that vB_EfaS-271 is safe for mammalian cells, and it is efficient in deceasing number of viable *E. faecalis*. However, they also suggest that conditions of high m.o.i. (10) may cause more rapid selection of bacteria resistant to this phage than low (0.0001) m.o.i. conditions.

### 3.3. Appearance of Phage-Resistant Bacteria

Results presented in Section 3.1 and Section 3.2 suggested that bacteria resistant to vB_EfaS-271 phage can appear. To test frequency of this appearance and efficiency of selection of resistant *E. faecalis*, we have performed experiments in which bacterial cultures were infected with the tested virus at various m.o.i., and bacterial growth, viability of cells, and fractions of phage-resistant bacteria were determined. 

**Figure 4:**
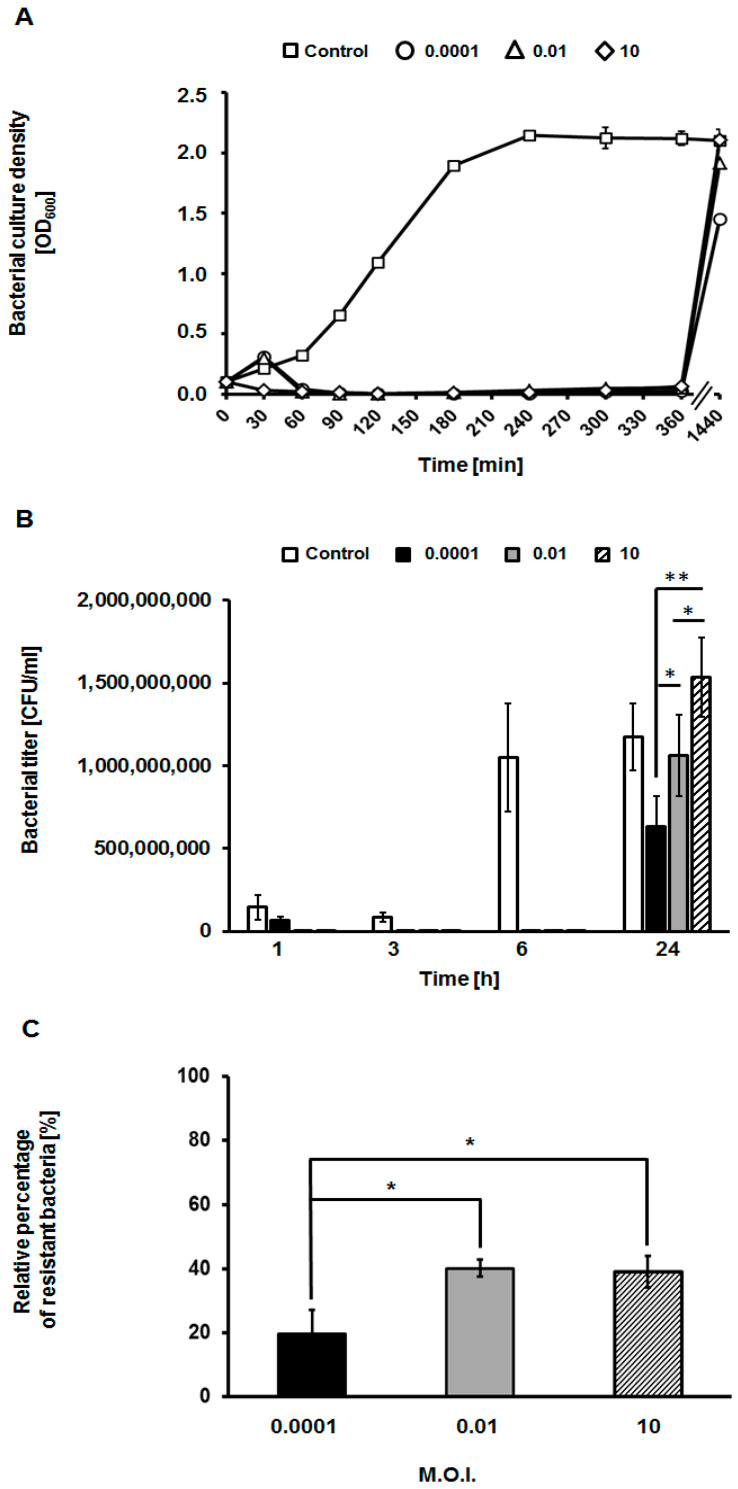
The lysis profile of host bacteria after infection of *E. faecalis* 271 with bacteriophage vB_EfaS-271 at different m.o.i. (0.0001, 0.01, and 10) (**A**,**B**), and the efficiency of formation of phage-resistant bacterial mutants during the infection process after 24 h (**C**). The results are presented as bacterial culture density (OD_600_) (**A**), number of bacterial cells surviving the phage infection per 1 mL (CFU/mL) (**B**), and percentage of resistant bacteria among survivors of the vB_EfaS-271 infection, after one passage without contact with phages (**C**). As a negative control, *E. faecalis* 271 host culture was inoculated with TSB medium instead of the tested virus. The results are shown as the mean values ± SD from three biological experiments. Note that in some cases (panel A), error bars are smaller than sizes of symbols. Significant differences between results obtained for control (no infection) and particular variants of phage infection experiments are marked by asterisks at *p* < 0.05 (*) or *p* < 0.01 (**).

When monitoring growth curves, it was clear that when measuring density of cultures, bacterial growth was rapidly (within 60 min) inhibited by the phage at all tested m.o.i. values (0.0001, 0.01, and 10) (Figure 4A). The growth was negligible until 6 h after infection. However, it was restored next day under all experimental conditions (time point 1440 min., i.e., 24 h). On the other hand, when determining number of viable cells by plating, it was evident that selection of resistant bacteria was more efficient at high m.o.i. (10), than at low m.o.i. (0.01 and 0.0001) at 24 h (Figure 4B). If percent of resistant bacteria was assessed after one passage of growth in the absence of phage vB_EfaS-271, fractions of phage-resistant cells were higher in experiments with m.o.i. of 0.01 and 10 than that determined at m.o.i. of 0.0001 (Figure 4C). These results indicated that selection of vB_EfaS-271 phage-resistant bacteria was more efficient at high m.o.i. than at low m.o.i.

## 4. Discussion

Since development of phage therapy against *E. faecalis* appears to be important to establish effective treatments of infections by antibiotic-resistant strains of this bacterium [1,2,3,4,5,6,7,8,9,10,11,12], and biofilm formation on catheters is one of the most difficult medical challenges caused by this species [13], we aimed to test efficiency of recently isolated and described bacteriophage vB_EfaS-271 [26] in killing *E. faecalis* cells included in biofilms formed on Foley silicone catheters. We found that vB_EfaS-271 is able to efficiently reduce number of viable bacterial cells under such conditions (Figure 1), providing evidence for its possible use in medical practice to either prevent colonization of catheters by *E. faecalis* or eradication of this bacterium from already formed biofilms in these medical devices. Nevertheless, after 24-h incubation under experimental conditions, appearance of phage-resistant bacteria was found, particularly when high m.o.i. (10) was used.

When testing effects of bacteriophage vB_EfaS-271 on mammalian cells (BALB/c3T3 mouse fibroblasts), neither toxicity nor changes in cell morphology could be observed (Figure 2 and Figure 3). This indicated that the investigated phage is safe for mammalian cells. However, efficiency of protection of mouse fibroblasts against *E. faecalis* by phages was higher at lower m.o.i., while appearance of phage-resistant bacteria was more rapid at higher m.o.i (Figure 2). These results again suggested that selection of phage-resistant bacteria was more effective under conditions when one bacterial cell was infected by several virions rather than under conditions of rare infection events in cell population. Such a scenario was confirmed in experiments where appearance of phage-resistant *E. faecalis* was investigated in 24-h long experiments (Figure 4).

We suggest that more efficient selection of vB_EfaS-271-resistant bacteria at high m.o.i. may result from population dynamics. Namely, according to the theory of evolution, mutations appear randomly in populations of any organisms under certain environmental conditions. Therefore, one should assume that vB_EfaS-271-resistant *E. faecalis* cells appear at certain frequency. However, it is likely that a phage-resistant mutant, although viable, is at least slightly deficient in one physiological processes relative to wild-type cell. Therefore, in the absence of bacteriophages, such mutants are outgrown by wild-type bacteria. However, in the presence of viruses that can efficiently infect and kill bacterial cells, phage-resistant mutant are selected and can propagate efficiently, contrary to bacteria susceptible to infection. If so, when *E. faecalis* culture is infected with vB_EfaS-271 at high m.o.i. (i.e., under conditions where each cell is supposed to be infected by phage), only previously formed phage-resistant mutants can survive, and then they propagate effectively as multiplicated phages cannot infect them. On the other hand, under low m.o.i. conditions, only a small fraction of bacterial cell population is infected by the phage. Therefore, pre-existing phage-resistant mutants, which are at the same time slightly defective in at least one other feature relative to wild-type cells, at outgrown by phage-sensitive, non-infected bacteria, at the beginning of the experiment. Only when bacteriophages multiplicate efficiently in more and more susceptible cells, and number of such cells became drastically limited, the phage-resistant mutant can efficiently compete with their wild-type counterparts and appear as predominant bacteria in the population. Such a hypothesis may be corroborated by results presented in Figure 4C, where fraction of phage-resistant cells dropped after one passage without the presence of phage vB_EfaS-271, indicating that phage resistance is accompanied by weakness of at least one other physiological feature of the cell, making these mutants less competitive when the phage is absent in the culture. Therefore, any revertant mutants, with restored wild-type phenotype, would again effectively compete with phage-resistant mutants.

Although appearance of vB_EfaS-271-resistant mutants might suggest a limitation in the use of this virus in phage therapy, the impaired growth and lower competitiveness of such mutants implicates that combination of phage therapy with other anti-bacterial treatment(s) can still be effective. Moreover, such impaired competitiveness might suggest that vB_EfaS-271-resistant *E. faecalis* mutants could be eliminated by natural microbiome of patients.

## 5. Conclusions

Bacteriophage vB_EfaS-271 is safe for mammalian cells, not influencing their viability and morphology. It is effective in decreasing number of viable *E. faecalis* cells in biofilms formed on catheters, and in co-cultures with mouse fibroblasts. On the other hand, vB_EfaS-271-resistant mutants can be selected, especially under conditions of high m.o.i. Nevertheless, such mutants appear to be less competitive relative to wild-type cells, suggesting that they might be eliminated under natural conditions. Therefore, vB_EfaS-271 may be considered a promising virus for its potential use in phage therapy.

## Data Availability

Raw results are available from authors upon request.
